# Short-chain free fatty acid receptors FFA2/GPR43 and FFA3/GPR41 as new potential therapeutic targets

**DOI:** 10.3389/fendo.2012.00111

**Published:** 2012-10-02

**Authors:** Trond Ulven

**Affiliations:** Department of Physics, Chemistry and Pharmacy, University of Southern DenmarkOdense, Denmark

**Keywords:** 7TM receptors, free fatty acids, GPCR, inflammation, metabolic diseases, short-chain fatty acids, type 2 diabetes

## Abstract

The deorphanization of the free fatty acid (FFA) receptors FFA1 (GPR40), FFA2 (GPR43), FFA3 (GPR41), GPR84, and GPR120 has made clear that the body is capable of recognizing and responding directly to nonesterified fatty acid of virtually any chain length. Colonic fermentation of dietary fiber produces high concentrations of the short-chain fatty acids (SCFAs) acetate, propionate and butyrate, a process which is important to health. The phylogenetically related 7-transmembrane (7TM) receptors free fatty acid receptor 2 (FFA2) and FFA3 are activated by these SCFAs, and several lines of evidence indicate that FFA2 and FFA3 mediate beneficial effects associated with a fiber-rich diet, and that they may be of interest as targets for treatment of inflammatory and metabolic diseases. FFA2 is highly expressed on immune cells, in particular neutrophils, and several studies suggest that the receptor plays a role in diseases involving a dysfunctional neutrophil response, such as inflammatory bowel disease (IBD). Both FFA2 and FFA3 have been implicated in metabolic diseases such as type 2 diabetes and in regulation of appetite. More research is however required to clarify the potential of the receptors as drug targets and establish if activation or inhibition would be the preferred mode of action. The availability of potent and selective receptor modulators is a prerequisite for these studies. The few modulators of FFA2 or FFA3 that have been published hitherto in the peer-reviewed literature in general have properties that make them less than ideal as such tools, but published patent applications indicate that better tool compounds might soon become available which should enable studies critical to validate the receptors as new drug targets.

## Introduction

Fatty acids, mostly in esterified form, make up a fundamental component in biology. It has long been recognized that certain nonesterified fatty acids are involved in regulation of metabolism and the immune system, for example, by conversion of arachidonic acid to potent signaling substances such as prostaglandins and leukotrienes. The realization that saturated or unsaturated free fatty acids (FFAs) of virtually any length can act directly as signaling molecules through cell surface receptors is however much more recent, and came with the identification of the 7-transmembrane receptors FFA1, FFA2, FFA3, GPR84, and GPR120, all activated by FFAs of various chain length.

In 1997, the genes *GPR40, GPR41, GPR42*, and *GPR43* were discovered in the course of a search for novel subtypes of the unrelated galanin receptor (Sawzdargo et al., 1997). The deorphanizations of the corresponding receptors GPR40, GPR41, and GPR43 were reported in 2003, when it became clear that they all respond to FFAs (Briscoe et al., 2003; Brown et al., 2003; Kotarsky et al., 2003; Le Poul et al., 2003; Nilsson et al., 2003), and the receptors were later renamed to FFA1, FFA3, and FFA2, respectively (http://www.iuphar.org/). *GPR42* was first believed to be an inactive pseudogene, but was recently found to be a functional polymorph of *GPR41* with 98% overall identity and 100% identity in the transmembrane (TM) domains (Liaw and Connolly, 2009). Subsequently, the phylogenetically distinct FFA receptors GPR84 and GPR120 have been identified (Hirasawa et al., 2005; Wang et al., 2006).

FFAs are generally divided into short-chain fatty acids (SCFAs), consisting of 1–6 carbon atoms, medium-chain fatty acids (MCFAs), with 7–12 carbon atoms, and long-chain fatty acids (LCFAs), with more than 12 carbon atoms. FFA1 (GPR40) is highly expressed in the pancreatic β-cells and is activated by saturated and unsaturated LCFAs and to a less extent by MCFAs, resulting in enhancement of glucose-stimulated insulin secretion, and was thus identified as a new potential target for treatment of type 2 diabetes (Itoh et al., 2003). This observation has attracted much attention, and several series of FFA1 ligands have been identified (Briscoe et al., 2006; Garrido et al., 2006; Song et al., 2007; Christiansen et al., 2008, 2010, 2011, 2012; Tan et al., 2008; Tikhonova et al., 2008; Humphries et al., 2009; Negoro et al., 2010, 2012; Sasaki et al., 2011; Houze et al., 2012; Mikami et al., 2012), of which the most advanced compound has demonstrated good efficacy in phase II studies and currently undergoes phase III clinical trials (Burant et al., 2012). GPR84 and GPR120 respond to MCFAs and LCFAs, respectively. These receptors have so far received less attention than FFA1 as potential drug targets, even though activation of GPR120 is associated with release of glucagon-like peptide-1 (GLP-1), increased insulin sensitivity and repression of macrophage-induced inflammation (Hirasawa et al., 2005; Oh et al., 2010). The recent finding that dysfunctional GPR120 lead to obesity in both mouse and human is expected to attract more attention to this receptor (Ichimura et al., 2012). Only a few ligands with moderate potency have until now been published on GPR84 and GPR120 (Briscoe et al., 2006; Wang et al., 2006; Suzuki et al., 2008). The first potent and selective GPR120 agonist was disclosed only recently (Shimpukade et al., 2012). In contrast to the other fatty acid receptors, FFA2 and FFA3 are activated by SCFAs. The receptors are phylogenetically related to FFA1, and pharmacological profiles of the three receptors have been thoroughly discussed in several excellent reviews (Brown et al., 2005; Covington et al., 2006; Milligan et al., 2006; Hirasawa et al., 2008; Stoddart et al., 2008b; Ichimura et al., 2009; Wellendorph et al., 2009; Hudson et al., 2011). This review will focus on the potential roles of the SCFA receptors FFA2 and FFA3 as targets for discovery of new therapeutics, and the currently known ligands for the receptors will be examined.

## The potential roles of FFA2 and FFA3 as drug targets

FFA2 and FFA3 were deorphanized simultaneously by three independent groups (Brown et al., 2003; Le Poul et al., 2003; Nilsson et al., 2003). The receptors are activated by high micromolar or millimolar concentrations of SCFAs, most notably acetate, propionate and butyrate, which are produced in total concentrations up to above 100 mM by colonic fermentation of dietary fiber (Topping and Clifton, 2001). FFA3 responds somewhat more strongly to the longer SCFAs, thus, formic acid (C1) and acetic acid (C2) exhibit higher potency at the human FFA2, whereas valeric acid (C5) and caproic acid (C6) have higher potency at hFFA3 (Brown et al., 2003; Le Poul et al., 2003). Although FFA2 and FFA3 were deorphanized about at the same time as FFA1, they have until now received less attention as drug discovery targets. One likely reason for this is that the indications that can be targeted by FFA2 and FFA3 have been less clear-cut than for FFA1.

### FFA2

FFA2 is expressed most highly in immune cells such as peripheral blood mononuclear cells (PBMC) and polymorphonuclear cells (PMN) with an especially high expression found in neutrophils (Brown et al., 2003; Le Poul et al., 2003; Nilsson et al., 2003). Several studies have demonstrated that FFA2 mediates the chemotactic effects of SCFAs on neutrophils (Le Poul et al., 2003; Maslowski et al., 2009; Sina et al., 2009; Vinolo et al., 2011). These observations suggested that FFA2 could be of interests for treatment of diseases associated with an excessive or defect neutrophil response, such as inflammatory bowel disease (IBD) or alcoholism-associated immune depression.

A high intake of fiber is associated with decreased risk of and beneficial effects on the IBDs ulcerative colitis and Crohn's disease (Hou et al., 2011). The recent observation that FFA2-deficient mice show exacerbated or unresolving inflammation in models of colitis, arthritis and asthma, and that germ-free wild-type mice, unable to convert fiber to SCFAs, show similarly exacerbated inflammatory conditions, indicate that SCFAs and FFA2 represents a link between a fiber-rich diet and its beneficial effects on the immune system and inflammation (Maslowski et al., 2009). These observations suggest that FFA2 agonists could be of interest for the treatment of IBD. On the other hand, FFA2-knockout mice exhibited diminished inflammatory response to dextrane sodium sulfate-induced colitis, a common model of IBD, and in particular exhibited reduced infiltration of PMNs (Sina et al., 2009). These results indicate that antagonists of FFA2 could have a role in treatment of intestinal inflammation. PMNs however also protect against bacterial infection or translocation, and there is a risk that FFA2 antagonists might interfere with these protective effects. Thus, although a link between FFA2 and IBD has been established, it is at present unclear if agonists or antagonists of FFA2 would be the preferred treatment. A recent study found elevated expression of FFA2 and FFA3 in fetal membranes and placenta after onset of labor, and that treatment of amnion explants with LPS significantly increased FFA2 expression, whereas co-treatment with LPS and propionate reduced the inflammatory response (Voltolini et al., 2012). These results suggest that FFA2 agonists could reduce maternal and fetal inflammation and might counteract preterm labor triggered by this.

It is known that SCFAs have tumor suppressive properties, effects that have been explained e.g., by inhibition of histone deacetylase (Medina et al., 1997). A recent report shows that FFA2 expression frequently is reduced or lost in colon cancer cells (Tang et al., 2011). Restoration of FFA2 expression in the tumor cells followed by propionate treatment induced G0/G1 cell cycle arrest, activated caspases, upregulated p21, decreased cyclin D3 and cyclin dependent kinases 1 and 2, and increased apoptotic cell death (Tang et al., 2011). The results suggest that FFA2 functions as a tumor suppressor and provides a possible mechanism for the putative connection between a high-fiber diet and lower incidence of colon cancer.

FFA2 has also been proposed to play a role in regulation of appetite and metabolism (Sleeth et al., 2010). A fiber-rich diet is linked to lower body weight and lower incidence of diabetes (Psaltopoulou et al., 2010), and some evidence indicates that SCFAs produced by colonic fermentation of fiber may be responsible for this through FFA2 (Sleeth et al., 2010). High-fiber diets are associated with increased levels of peptide YY (PYY), a hormone known to decrease appetite (Karra and Batterham, 2010). FFA2 is found to be expressed in enteroendocrine L cells, responsible for PYY secretion (Karaki et al., 2006, 2008). The L cells are also responsible for GLP-1 secretion, a potent anorectic incretin hormone which also regulate insulin secretion from pancreatic β-cells and increase insulin sensitivity in target tissue. It was recently demonstrated that expression of FFA2 and FFA3 is enriched in L cells and that FFA2 mediate SCFA-promoted GLP-1 release from mixed colonic cultures *in vitro* (Tolhurst et al., 2012). These observations indicate that FFA2 agonists can have potential as therapeutics for treatment of type 2 diabetes and related metabolic conditions. FFA2 is also expressed in the murine pancreatic β-cell line MIN6 and isolated mouse islets (Kebede et al., 2009; Halpern et al., 2012), but the role of the receptor in β-cells is still unclear.

Also related to the effects of dietary fiber on body weight are the observations that FFA2 is expressed in murine adipose tissue and that acetate and propionate affect adipogenesis and adipocyte differentiation through FFA2 in mice (Hong et al., 2005). Activation of FFA2 furthermore leads to inhibition of lipolysis and suppression of plasma FFAs without the flushing associated with nicotinic acid, and the receptor may thus represent a target for treatment of dyslipidemia (Ge et al., 2008). The beneficial effects on high density lipoprotein (HDL) levels observed after moderate alcohol consumption might be at least partially a consequence of acetate, a metabolite of ethanol, on FFA2. A recent study found that FFA2-deficient mice on a high-fat diet exhibited improved glucose control, reduced body fat mass and increased insulin sensitivity, indicating that FFA2 antagonists might be of interest for treatment of metabolic disorders (Bjursell et al., 2011). Another recent study found that inulin-type fructans, known to counteract high-fat diet-induced obesity and other metabolic disorders, inhibited the FFA2 overexpression in adipose tissue normally seen with high-fat diets (Dewulf et al., 2011).

Thus, several lines of evidence link FFA2 to food intake, body weight and metabolic disorders such as type 2 diabetes. More research is however required to firmly conclude that FFA2 is an attractive target for these conditions and clarify if agonists or antagonists will be the preferred mode of treatment.

### FFA3

FFA3 was deorphanized independently by two groups (Brown et al., 2003; Le Poul et al., 2003). Both groups found FFA3 mRNA broadly expressed in various tissue, including pancreas, PBMC, spleen and adipose. Brown and co-workers found especially high levels of FFA3 mRNA in human adipose tissue and confirmed high expression in white adipose tissue with immunohistochemistry using an antibody specific for hFFA3 and hGPR42 (Brown et al., 2003). Subsequently, expression of FFA3 in both human and mouse adipose tissue was confirmed and it was found that SCFAs stimulate production of the anorexigenic hormone leptin in primary mouse white adipose tissue through FFA3 (Xiong et al., 2004). Hong and co-workers (2005) were however unable to detect FFA3 in murine adipose tissue or 3T3-L1 adipocytes, but found high levels of FFA2. They found that blocking FFA2 expression with siRNA blocked adipocyte differentiation, and concluded that the effects of SCFAs on adipocytes in mice are mediated through FFA2 rather than FFA3 (Hong et al., 2005). This conclusion was supported by Zaibi and co-workers (2010), who also failed to detect FFA3 mRNA in mouse adipose tissue and furthermore observed that FFA2 expression was consistently reduced in adipose tissue of FFA3 knockout mice compared to wild type mice. They confirmed SCFA-promoted leptin secretion in wild-type mice and reduced secretion in the FFA3 knockout mice, an effect which was explained by concomitant downregulation of FFA2 in the FFA3 knockouts (Zaibi et al., 2010). These observations underline the need for selective tool compounds in the further study of these receptors.

Like FFA2, FFA3 is expressed in enteroendocrine cells secreting the satiety hormone PYY and may be implicated in its release (Karaki et al., 2006). The receptor is also, like FFA1 and FFA2, expressed in pancreatic β-cells (Kebede et al., 2009; Halpern et al., 2012), but its role in these cells is at present unclear, although a patent application indicates that FFA3 agonists inhibit insulin secretion (see below). Samuel and co-workers found FFA3-knockout mice to be significantly leaner than wild-type mice. The difference disappeared in germ-free mice, indicating that the effect depends on SCFAs produced by fermentation. Deletion of FFA3 was associated with reduced secretion of PYY, but without affecting the amount of chow consumed by the mice. This led the authors to suggest that the reduction in weight is a result of the increased gut motility by the lower PYY level, leading to a reduced uptake of SCFAs (Samuel et al., 2008). The lean phenotype of FFA3 knockout mice is inconsistent with its suggested role as mediator of leptin secretion. However, Zaibi et al. (2010) mention unpublished observations that male FFA3 knockout mice are obese rather than lean.

**Figure 1:**
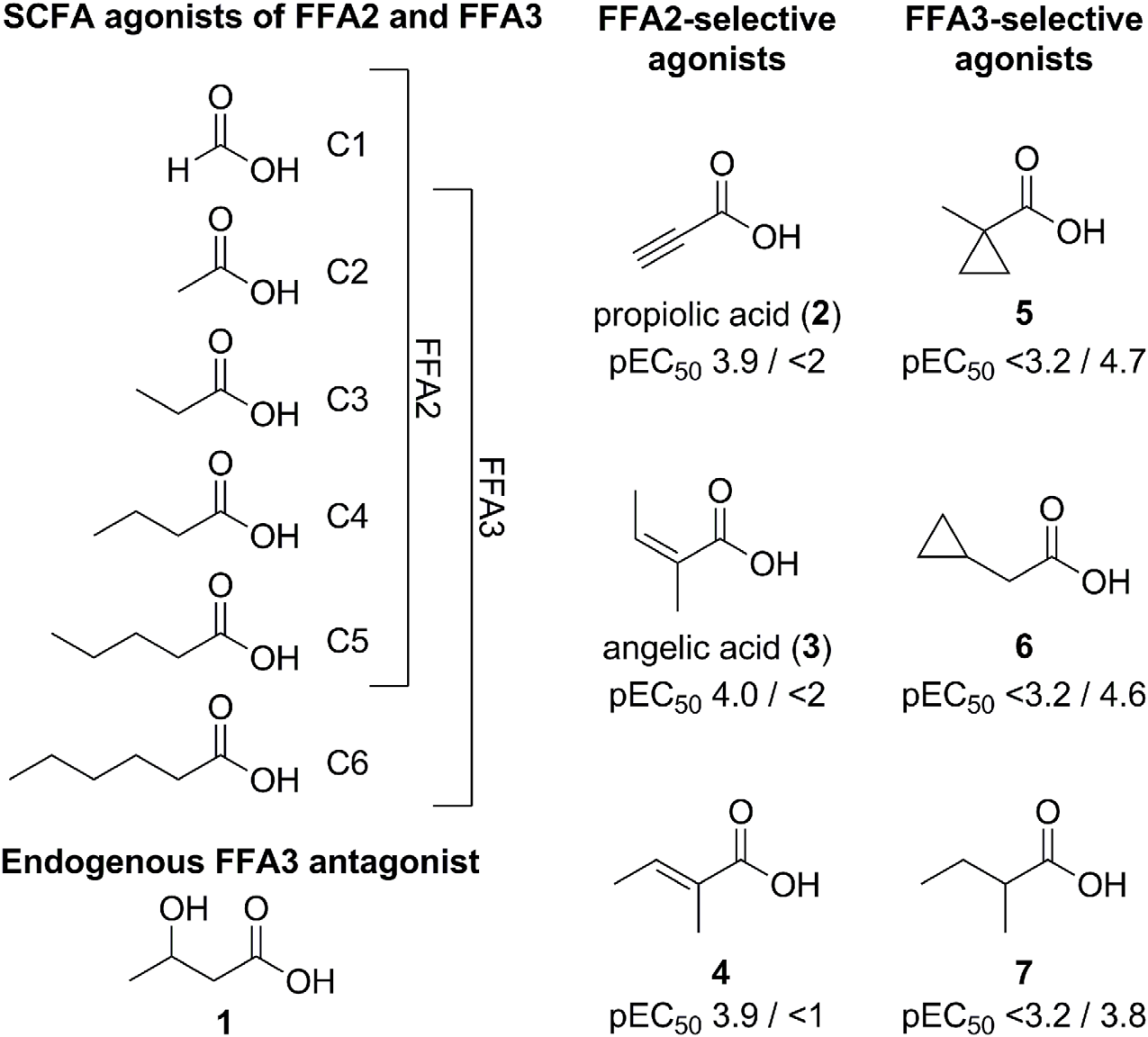
**SCFAs and other small carboxylic acid modulators of FFA2 and FFA3.** pEC_50_ values of **2–7** for FFA2/FFA3 are from a dynamic mass redistribution assay (Schmidt et al., 2011).

SCFAs and ketone bodies are found to regulate the sympathetic nervous system directly through FFA3 at the sympathetic ganglion. SCFAs stimulate the sympathetic outflow by activating FFA3, whereas β-hydroxybutyrate (**1**, Figure 1), a metabolite produced during the ketogenic state of starvation or diabetes, antagonize FFA3 and thereby suppress the sympathetic nervous system (Kimura et al., 2011). Thus, FFA3 appears to contribute to sympathetic activation in the fed state when glucose is the main fuel and abundant production of SCFAs by colonic fermentation of dietary fiber take place, whereas the receptor contributes to energy conservation under ketogenic conditions during fasting. This observation implicates that FFA3 agonists could represent a potential treatment of obesity.

Several studies have connected FFA3 with metabolic regulation and energy expenditure, but expression in adipose tissue is controversial and the overall function of the receptor is still unclear. Further research is therefore required to clarify the picture and selective tool compounds would be highly valuable to this research.

## Ligands for FFA2 and FFA3

Although it is a decade since FFA2 and FFA3 were deorphanized, few ligands have been reported for the receptors. The initial publications disclosed EC_50_ values for SCFAs in the high micromolar and low millimolar range, corresponding to physiological concentrations (Brown et al., 2003; Le Poul et al., 2003; Nilsson et al., 2003). The potency rank order of SCFAs for FFA2 is acetate (C2) ~ propionate (C3) > butyrate (C4) > valerate (C5) > formate (C1). The corresponding rank order for FFA3 was somewhat different, with propionate (C3) ~ butyrate (C4) ~ valerate (C5) > acetate (C2) > caproate (C6). Thus, FFA2 has a preference for the shorter SCFAs, whereas FFA3 prefer the longer SCFAs, with propionate (C3) being among the most potent SCFA for both receptors (Figure 1).

**Figure 2:**
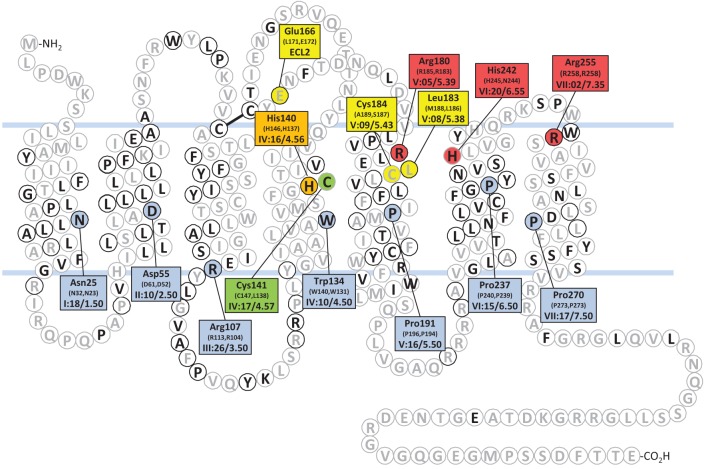
**Snake plot of the human FFA2.** Residues with black letters are conserved between hFFA2 and hFFA3. Residues in black circles are conserved between hFFA2 and hFFA1. Highlighted hFFA2 residues are identified in the connected squares, the corresponding residues of hFFA3 and hFF1 are given in parenthesis, and Schwartz-Baldwin (Rosenkilde et al., 2010) and Ballesteros-Weinstein (Ballesteros and Weinstein, 1995) notations for TM residue positions are indicated. The most conserved residue of each helix throughout family A of the 7TM receptors are blue. The two arginines and the histidine critical for recognition of SCFAs in FFA2 (R180, H242, R255) and FFA3 (R185, H245, R258) are red and the histidine important for SCFA recognition in FFA2 (H140) and critical in FFA3 (H146) is orange (Stoddart et al., 2008a). The three residues that reverse the selectivity for **4** and **5** when swapped (FFA2: E166L, L183M, C184A; FFA3: L171E, M188L, A189C) are yellow (Schmidt et al., 2011). The cysteine that enables hFFA2 to be activated by longer FFAs when mutated to glycine is green (Hudson et al., 2012).

By site-directed mutagenesis, an arginine residue near the top of TM helix 5 and another arginine at the top of TM 7 were found to be critical for the recognition of SCFAs for both FFA2 (Arg180^5.39^, Arg255^7.35^) and FFA3 (Arg185^5.39^, Arg258^7.35^) (Figure 2) (Stoddart et al., 2008a). Two corresponding residues in FFA1 (Arg183^5.39^ and Arg258^7.35^) are likewise critical for recognition of LCFAs (Sum et al., 2007). Thus, the two arginines function as conserved anchoring residues for the fatty acid carboxylate group throughout the FFA1-3 receptor subfamily. In addition, the mutation His(VI:20/6.55)Ala in hFFA2 or hFFA3 eliminated the response to SCFAs (Stoddart et al., 2008a). The corresponding residue in FFA1, Asn244^6.55^, is also critical for FFA recognition. Furthermore, mutation of His146^4.56^ to alanine eliminated SCFA response in FFA3, whereas the corresponding mutations in FFA2 (His140^4.56^) or FFA1 (His136^4.56^) only reduced the response to SCFAs and LCFAs, respectively (Sum et al., 2007; Stoddart et al., 2008a). Thus, the recognition of the fatty acid carboxylate group appears to take place by a conserved mechanism in FFA1, FFA2, and FFA3.

The low potency and selectivity of the SCFAs on FFA2 and FFA3 make them difficult starting points for optimization and generally unsuitable as pharmacological tool compounds. However, when their size is taken into account, the potency of acetate and propionate on these receptors is quite impressive and ligand efficiency calculations indicate that it is unrealistic to expect higher potency without significantly increasing the size of the compounds (Schmidt et al., 2011). To explore the structure-activity relationships around the SCFAs on FFA2 and FFA3, a diverse selection of small carboxylic acids with lipophilic tails, including branched, cyclic and unsaturated, were selected and characterized on hFFA2 and hFFA3. None of the compounds exhibited higher potency than the most potent SCFAs, but the study resulted in the elucidation of a general rule for predicting the selectivity of small carboxylic acids for FFA2 or FFA3: Ligands with substituted sp^3^-hybridized α-carbons preferentially activate FFA3, while ligands with sp^2^- or sp-hybridized α-carbons prefer FFA2 (Schmidt et al., 2011). For example, propiolic acid (**2**), angelic acid (**3**) and (*E*)-2-methylcrotonic acid (**4**) are all at least 10–100-fold selective for hFFA2 over hFFA3, whereas 1-methylcyclopropanecarboxylic acid (**5**), cyclopropylacetic acid (**6**) and 2-methylbutyric acid (**7**) have distinct selectivity for hFFA3 over hFFA2 (Figure 1). The compounds were inactive on the Arg(V:05/5.39)Ala and Arg(VII:01/7.35)Ala mutants, confirming interaction at the orthosteric binding site. Moreover, by swapping three nonconserved amino acid residues between FFA2 and FFA3 (Figure 2), the selectivity of **4** and **5** was inverted (Schmidt et al., 2011). This thorough understanding of the binding site interaction and selectivity of the small carboxylic acids makes a good basis for using these structures in the discovery of selective FFA2 and FFA3 ligands, for example in a fragment-based approach.

The bovine FFA2 is less sensitive to the shortest SCFAs and responds most strongly to caproic acid (C6), probably reflecting a digestion adapted for non-digestible carbohydrates and higher levels of SCFAs (Hudson et al., 2012). Mutation of Cys141^4.57^ in hFFA2 (Figure 2) to the Gly corresponding to the bovine receptor transferred a similar ligand selectivity to the human receptor. By introducing a second H242Q mutation in hFFA2, the receptor lost its response to SCFAs, which was taken advantage of in the construction of a receptor activated solely by a synthetic ligand (RASSL) of FFA2 (Hudson et al., 2012).

**Figure 3:**
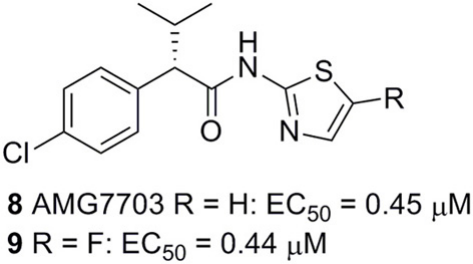
**Selective allosteric agonists of FFA2 (Amgen).** Activities from an aequorin-based calcium assay are given (Lee et al., 2008).

Amgen has described two closely related phenylacetamides as allosteric agonists of FFA2 (Figure 3). The compounds are completely selective for FFA2 over FFA3 and FFA1, produce full agonistic response alone, and act in a positively cooperative fashion with acetate or propionate. Both compounds were shown to activate Gα_i_ and Gα_q_ pathways and to inhibit lipolysis in adipocytes (Lee et al., 2008). The racemic compound (rac-**8**) was identified by high-throughput screening, of which the (S)-enantiomer AMG7703 (**8**, Figure 3) was found to be responsible for the activity. Mutational studies have been performed to identify the binding mode of **8** and related compounds, however, residues that interfere with **8** without significantly affecting acetate or propionate activity have not been identified and the exact binding of the ligands remains somewhat unclear (Lee et al., 2008; Smith et al., 2011). It is however clear that the binding of **8** does not depend on Arg180^5.39^ and Arg255^7.35^, both of which serve as critical carboxylic acid anchoring points for the SCFAs (Swaminath et al., 2010; Smith et al., 2011) Interestingly, replacement of ECL2 of FFA2 by the corresponding sequence from FFA3 did not affect the potency of **8** or propionate, but resulted in reduced efficacy and abolishment of the cooperative effect between the two compounds (Smith et al., 2011). Attempts to optimize these compounds have been made, however, despite the synthesis of a large number of analogs with modifications introduced in all parts of the structure, it has turned out difficult to significantly improve their relatively moderate potency (Wang et al., 2010; Smith et al., 2011). Besides insufficient potency, the compounds also suffer from moderate solubility and poor pharmacokinetic properties (Wang et al., 2010), and are therefore of limited use as tool compounds for *in vitro* and *in vivo* studies.

**Figure 4:**
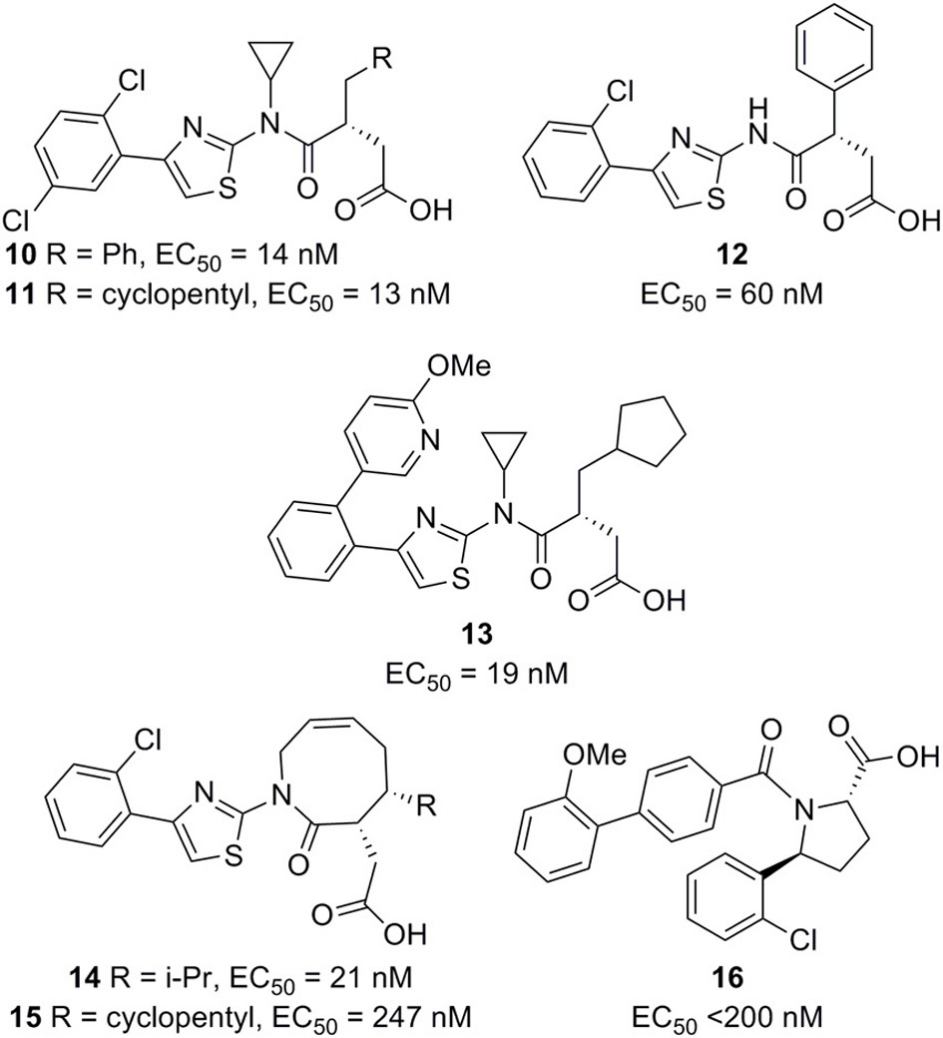
**FFA2 agonists (Euroscreen).** The EC_50_ values are from GTPγS binding in a scintillation proximity assay (Hoveyda et al., 2010, 2011a,b,c,d).

No FFA2 or FFA3 modulators with desirable potency and properties have yet been reported in peer reviewed journals, but FFA2 ligands of interest have been described in recent patent applications. Euroscreen has patented a compound series of FFA2 agonists disclosing compounds with potency down to EC_50_ = 13 nM (Figure 4) and claims their use in treatment of metabolic disorders (Hoveyda et al., 2010). A representative compound (**12**) increases glucose uptake in 3T3-L1 cells in a dose dependent manner. The increased glucose uptake was slight but significant at 1 μM and almost doubled at 30 μM. The phenylacetamide **8** was not observed to increase glucose uptake in this assay. Furthermore, **12** dose-dependently inhibited lipolysis in isolated adipocytes. Again, **8** showed no effect in the assay. Compound **12** was also found to increase glucose uptake in isolated adipocytes by 100% already at 1 μM concentration. Another representative (**13**) was found to increase GLP-1 secretion from NCI-H716 cells (Hoveyda et al., 2010). Euroscreen has moreover claimed the compound series for treatment of gastrointestinal disorders and inflammatory diseases, including IBD, and has found representatives to decrease colonic contractility and motility and to decrease TNF-α and IL-6 release from PBMCs stimulated with LPS (Hoveyda et al., 2011a,b). A series of constrained lactam analogs has appeared in a recent patent application (Hoveyda et al., 2011d), including compounds with EC_50_ down to 21 nM (**14**) and one representative (**15**) demonstrated in concentrations from 0.1 to 30 μM to significantly and dose dependently increased GLP-1 secretion from a rat lower intestinal cell preparation. A separate series of 5-aryl-2-acylpyrrollidinecarboxylic acid FFA2 agonists also patented by Euroscreen contains several members with EC_50_<200 nM. Of these, **16** was found to inhibit isoprenaline-induced lipolysis in rat adipocytes and reduce the blood glucose level in *ob/ob* mice after glucose challenge following 28 days of chronic treatment (Figure 4) (Hoveyda et al., 2011c).

**Figure 5:**
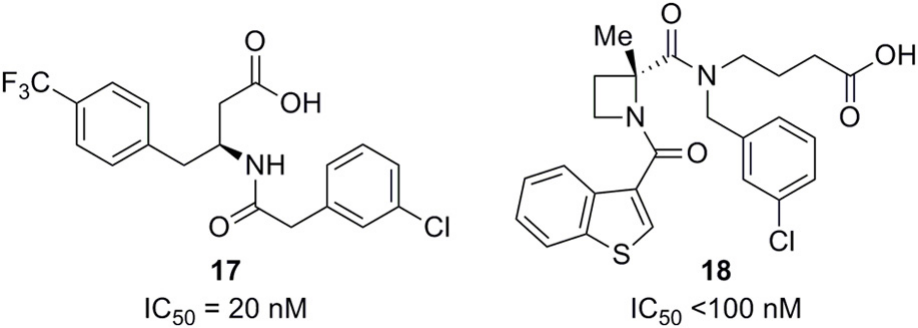
**FFA2 antagonists (Euroscreen and Galapagos).** The IC_50_ value of **17** is from a GTPγS assay in CHO cells expressing FFA2 using propionate (600 μM) as agonist (Brantis et al., 2011). The IC_50_ value of **18** reflects a calcium mobilization assay and a GTPγS assay (Saniere et al., 2012).

Euroscreen has furthermore patented a series of FFA2 antagonists for treatment or prevention of inflammatory, gastrointestinal and metabolic disorders, including **17**, which was disclosed with IC_50_ = 10 nM in a calcium-based assay and 20 nM in a GTPγS assay (Figure 5). Homologous competition binding studies with tritiated **17** exhibited pIC_50_ ~8 in a human FFA2 recombinant cell line and pIC_50_ ~6 in neutrophils. The radiotracer was also displaced from the FFA2 transfected cells by propionate with pIC_50_ ~2.5, suggesting orthosteric interaction (Brantis et al., 2011). This observation demonstrates that the orthosteric binding site can accommodate ligands that are larger than valeric acid (C5, Figure 1). It indeed appears reasonable to hypothesize that all ligands in Figures 4 and 5 act as orthosteric ligands and that their carboxylic acid residues are engaged in interactions similar to those of the endogenous SCFAs.

A series of azetidine FFA2 antagonists is claimed for treatment of inflammatory conditions and autoimmune, infectious, cardiometabolic, and proliferative diseases in a recent patent application from Galapagos (Saniere et al., 2012). The representative compound **18** exhibited IC_50_ below 100 nM in a calcium mobilization assay, a GTPγS binding assay, both using acetate at a concentration corresponding to EC_80_, and a neutrophil migration assay using acetate as chemoattractant.

**Figure 6:**
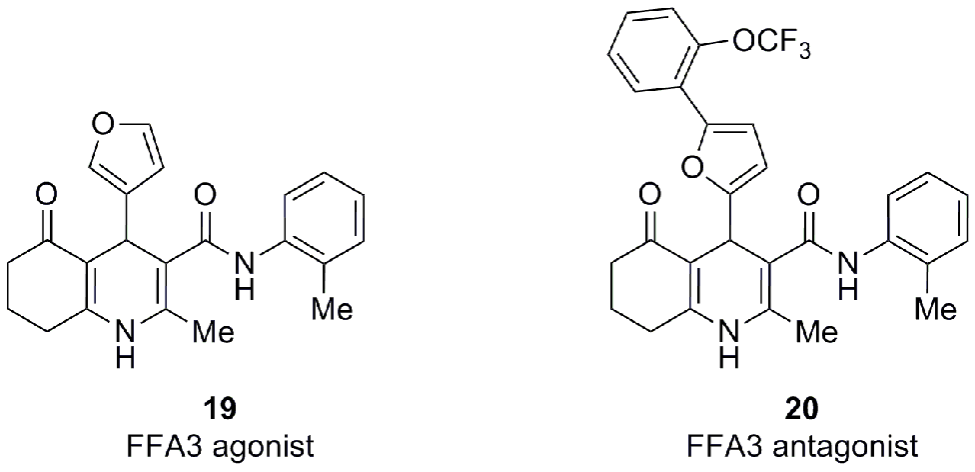
**FFA3 modulators (Arena) (Leonard et al., 2006)**.

There has been less activity directed toward development of selective FFA3 modulators. An early patent application from Arena claims methods and compounds related to FFA3 (Leonard et al., 2006). The application describes results showing that FFA3 is highly expressed in human and mouse pancreas, that the receptor is upregulated in *db/db* mice, compared to wild-type mice and leptin-deficient *ob/ob* mice, and that it is expressed in the insulin-producing β-cell lines NIT-1, βTC-6, and MIN6. Several compounds are disclosed as FFA3 agonists or antagonists. Cyclopropanecarboxylic acid, a mixed FFA2/FFA3 agonist with moderate selectivity for FFA3 (Schmidt et al., 2011), was found to inhibit insulin secretion from the murine insulinoma cell line MIN6. The patent application also discloses the structure of FFA3 agonists such as **19** (Figure 6) and FFA3 antagonists such as **20**, but no indication of their potencies is given. The FFA3 agonist **19** reverses the beneficial effect on oral glucose tolerance test of the GPR119 agonist B111 (AR231453) in mice, which might imply that agonism is not the preferred mode of action.

## Conclusion

It is clear that the intestinal microbiome has a significant influence on health (Kau et al., 2011). An illustrative example of this is provided by a recent study demonstrating that a lower number of colonic bacterial species is associated with higher body weight in pairs of lean and obese twins (Turnbaugh et al., 2009). A fiber-rich diet has long been established as a significant contributor to good health. The gut microbiome is responsible for the fermentation of dietary fiber, which leads to production of SCFAs and several beneficial health effects. Recent results suggest that the SCFA receptors FFA2 and FFA3 are implicated in several of these health effects.

FFA2 and FFA3 were deorphanized at the same time as FFA1, but have received comparably less attention as drug targets. Accumulating evidence however indicates that the receptors are of interest as potential targets for treatment of various conditions and diseases related to immunology and metabolism. FFA2 plays a role in certain immune diseases where neutrophils are implicated, such as ulcerative colitis and Crohn's disease. Both FFA2 and FFA3 have been implicated with weight regulation and metabolic diseases like type 2 diabetes by several lines of evidence, although some contradictory results blurs the picture for FFA3. Both receptors are expressed in the intestines and it was recently reported that FFA2 mediates SCFA-promoted GLP-1 release. Results are described in recent patent applications indicating that FFA2 agonists indeed promote GLP-1 release and that they furthermore increase glucose uptake in adipocytes, thus providing support for the notion that FFA2 agonists could be of interest for the treatment of type 2 diabetes. Although the prospects look interesting, additional research is necessary to firmly establish the receptors as drug targets and the mode of action for drug candidates. Potent and selective tool compounds will be required for such studies, and recent patent applications indicate that such tools might soon become available.

### Conflict of interest statement

The author declares that the research was conducted in the absence of any commercial or financial relationships that could be construed as a potential conflict of interest.
